# Age-Associated Differences in Recovery from Exercise-Induced Muscle Damage

**DOI:** 10.3390/cells13030255

**Published:** 2024-01-30

**Authors:** Donna Ching Wah Li, Stefan Rudloff, Henning Tim Langer, Kristina Norman, Catrin Herpich

**Affiliations:** 1 Department of Nutrition and Gerontology, German Institute of Human Nutrition Potsdam-Rehbrücke, 14558 Nuthetal, Germany; donna.li@dife.de (D.C.W.L.); catrin.herpich@dife.de (C.H.); 2 Institute of Nutritional Science, University of Potsdam, 14558 Nuthetal, Germany; 3 Department of Geriatrics and Medical Gerontology, Charité–Universitätsmedizin Berlin, Corporate Member of Freie Universität Berlin and Humboldt-Universität zu Berlin, 13347 Berlin, Germany; stefan.rudloff@charite.de; 4 Department of Medicine, Weill Cornell Medicine, New York, NY 10065, USA; htl4001@med.cornell.edu; 5 German Center for Cardiovascular Research (DZHK), Partner Site Berlin, 10785 Berlin, Germany

**Keywords:** aging, exercise, recovery, EIMD

## Abstract

Understanding the intricate mechanisms governing the cellular response to resistance exercise is paramount for promoting healthy aging. This narrative review explored the age-related alterations in recovery from resistance exercise, focusing on the nuanced aspects of exercise-induced muscle damage in older adults. Due to the limited number of studies in older adults that attempt to delineate age differences in muscle discovery, we delve into the multifaceted cellular influences of chronic low-grade inflammation, modifications in the extracellular matrix, and the role of lipid mediators in shaping the recovery landscape in aging skeletal muscle. From our literature search, it is evident that aged muscle displays delayed, prolonged, and inefficient recovery. These changes can be attributed to anabolic resistance, the stiffening of the extracellular matrix, mitochondrial dysfunction, and unresolved inflammation as well as alterations in satellite cell function. Collectively, these age-related impairments may impact subsequent adaptations to resistance exercise. Insights gleaned from this exploration may inform targeted interventions aimed at enhancing the efficacy of resistance training programs tailored to the specific needs of older adults, ultimately fostering healthy aging and preserving functional independence.

## 1. Introduction

Discernible structural and functional differences in aging skeletal muscle increase physical limitations and the risk of other morbidities. One such age-related change, sarcopenia, the loss of both muscle strength and size, is thought to be a primary contributor to the etiology of physical and functional decline in older adults. Given that skeletal muscle mass accounts for 40% of body mass, its preservation and health have profound consequences [1]. Resistance exercise (RE) is an effective method to offset age-associated deterioration. Eccentric exercise is also often recommended during rehabilitation or injury prevention [2,3,4,5]. However, the exposure of unaccustomed RE to naïve muscle induces exercise-induced muscle damage (EIMD). The prevalence of EIMD is associated with the high force eccentric component of a contraction, although it is generally accepted that EIMD is present with conventional RE consisting of both concentric, isometric, and eccentric actions. Other common models to induce EIMD include: downhill running [6,7], prolonged running [8], drop jumps [9,10], stretch-shortening cycle exercise [11], and cycling [12,13]. EIMD necessitates an appropriate recovery response, a fine-tuned cellular balance between the clearance of damaged cell debris and the remodeling and regeneration of the skeletal muscle. Central to the cumulative positive alterations by RE is adequate recovery which requires the occurrence of a series of systematic cellular events. Otherwise, long-term beneficial outcomes, such as hypertrophy, are hampered.

The adequacy of such recovery is likely impaired in older age. Especially in the face of EIMD, recovery is delayed or attenuated following an acute exercise challenge [14]. This impairment is likely both a cause and consequence of aging muscle. Considering RE in the maintenance of skeletal muscle health and function across the lifespan, characterization of the stepwise kinetics of muscle recovery from EIMD will add to the understanding of not only exercise recovery, but also the timelines pertaining to post-trauma recovery and rehabilitation [15]. Cellular mechanisms involved in the accruement and resolution of EIMD have been explored in young study participants [16], whereas less research has been conducted in older adults. Therefore, this review will analyze potential age-related deficits that participate in dysregulated recovery from EIMD, with particular regard to molecular mechanisms involving inflammation, extracellular matrix (ECM) remodeling, and lipid mediators. But first, the etiology and pathogenesis of EIMD will be dissected to visualize how each of these mechanisms fits into the bigger picture.

## 2. Exercise-Induced Muscle Damage

In this chapter, we briefly summarize the general origin and stepwise progression of EIMD.

### 2.1. Etiology and Cellular Dynamics of EIMD

An acute consequence of RE can be EIMD, especially when the exercise comprises high-volume, high-velocity and/or eccentrically biased muscle actions [17,18]. EIMD is a transient phenomenon characterized by structural and functional consequences that are present both immediately and up to ~14 days following the cessation of the initial exercise bout [19]. Symptoms of EIMD include muscle soreness, diminished force-producing capacity, ultrastructural disruptions, increases in intramuscular proteins in circulation, and swelling. These symptoms usually peak 12 to 48 h after exercise. The magnitude and duration of these symptoms vary and depend on the exercise protocol, muscle group and/or training status [20]. The current gold-standard indirect marker for assessing damage is reduced muscle force [21]. Other common measures such as delayed onset muscle soreness (DOMS) and circulating creatine kinase generally have large intra- and inter-individual variation and should only be used for indicating the presence of EIMD and not as a method to quantify its magnitude [21]. The effects of age on exercise-induced muscle damage are thoroughly reviewed here [22]. Although EIMD is a natural, positive stimulus for optimal muscular adaptations, ensuing mobility limitations may affect daily activity. The mechanisms of EIMD can be distilled into two phases: (i) the initial phase or primary damage resulting from mechanical stress during the exercise and (ii) the inflammatory phase or secondary damage that can aggravate tissue damage [20].

### 2.2. Primary Muscle Damage—Mechanical Stress

Primary muscle damage is associated with the overstretching of the sarcomeres, failure of excitation–contraction coupling, and disrupted ECM [23]. This is especially prominent during eccentric contractions. During an eccentric contraction, lower activation and recruitment of faster motor units induce greater mechanical stress on fewer muscle fibers and the non-uniform lengthening of sarcomeres, respectively [24,25,26]. The ‘popping sarcomere hypothesis’ posits the most significant length change in the weakest sarcomere, making it “pop” during a contraction [26]. Repeated contractions lead to the “popping” of the next weakest sarcomere. Collectively, this increases membrane permeability and disrupts the excitation–contraction coupling process [23,26,27]. Disruption of the cell membrane and components of the excitation–contraction coupling machinery can lead to aberrant calcium (Ca^2+^) movement into the cytoplasm, tilting calcium homeostasis [23]. Extracellular influx of Ca^2+^ activates the Ca^2+^ sensitive phospholipase–prostaglandin pathway and calpain proteolytic pathway for the removal of damaged proteins [28,29] and cell membrane breakdown, further increasing permeability and the leakage of intracellular components. At the same time, calpain cleaves cytoskeleton proteins located at the Z-disc regions. Excess Ca^2+^ is taken up by the mitochondria, furthering apoptosis or necrosis as the permeability of the inner mitochondrial membrane increases, causing the uncontrolled movement of Ca^2+^ into the intracellular space [28]. This increase may explain the increased passive tension of the muscle due to the inability of the muscle to relax properly [26,30]. ECM remodeling may also influence passive muscle stiffness as it separates from the myofiber [31]. The ECM may further play a role by modulating intracellular cascades to mediate transcription and excretion of molecules in muscle and neighboring cells [32,33]. It has been proposed that ECM damage initiates the inflammatory phase [31].

### 2.3. Secondary Muscle Damage—Inflammatory Phase

Delayed secondary muscle damage is linked to a highly controlled and temporally organized inflammatory response [34]. Inflammation is necessary for successful and efficient muscle regeneration and blocking the inflammatory process by anti-inflammatory modulators might result in maladaptation and impaired muscle hypertrophy in response to functional overload [35]. This phase begins with the rapid infiltration of neutrophils in the minutes to hours after exercise, activated, in part, by increased intracellular Ca^2+^ [33,36]. Neutrophils actively phagocytose and eliminate damaged tissues [37]. In young healthy humans, neutrophil levels are most pronounced at 24 h [38,39,40,41,42]. Other immune cells are also mobilized, including mast cells, neutrophils, T regulatory lymphocytes, eosinophils, and CD8 T lymphocytes [39]. In the following hours to days, blood monocytes are recruited and differentiated into tissue macrophages, replacing neutrophils [43,44]. Initially, macrophages of the M1 phenotype dominate to ingest debris and apoptotic neutrophils. This process along with the local production of pro-inflammatory cytokines (e.g., interleukin (IL)-1 beta, tumor necrosis factor alpha (TNFa)) by muscle cells triggers a phenotypic switch to the alternatively activated anti-inflammatory (M2) phenotype that facilitates muscle growth and regeneration [45,46]. Macrophages can remain in the tissue for up to two weeks [47].

### 2.4. Resolving Inflammation—Resolution Phase

This process must occur in a timely manner to protect against the unnecessary destruction of healthy muscle tissue [48]. Successful resolution requires the apoptosis of neutrophils, arrest of leukocyte infiltration, and macrophage efferocytosis of apoptotic cells [49,50]. This process is mediated, in part, by lipid mediators [51]. Several classes of mediators with anti-inflammatory and pro-resolving bioactivity actively promote the return to a non-inflamed state. This “braking” signaling is crucial in preventing prolonged inflammation. Possibly, the inability of aged muscle to resolve this itself may lead to the damage of healthy muscle tissue and deter long-term beneficial muscular adaptations. The aberrant resolution of inflammation may be one of the many contributing factors along the different stages of recovery to contribute to the deficits seen in aging.

## 3. Factors Contributing to Age-Related Impairment in Exercise Recovery

**Figure 1:**
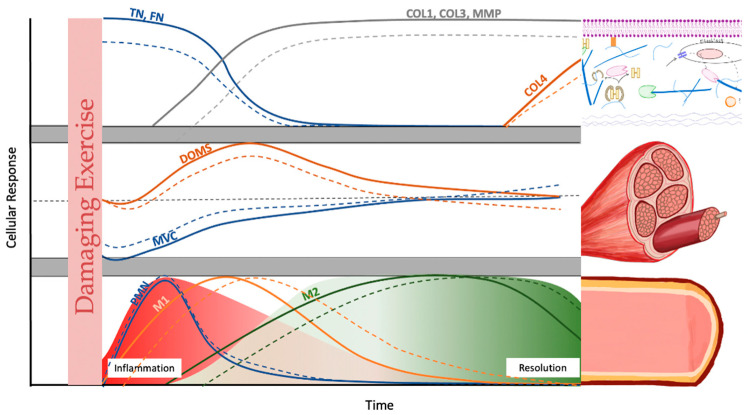
Schematic comparison of the cellular response kinetics of post damaging exercise in young (line) and old humans (dotted line): extracellular matrix (ECM) changes (upper panel), indirect markers (middle panel), and inflammatory expression (lower panel). Upper panel: Tenascin-C (TN) and fibronectin (FN) are part of the transitional matrix and are thought to direct early satellite cell movement. Subsequently, collagens (COL) 1 and 3 and their matrix metalloproteinases (MMPs) increase for ECM remodeling. COL 4 is, an important component of the basement membrane, is thought to be remodeled during later stages. Middle panel: Decreases in maximal voluntary contraction (MVC) are prevalent immediately following exercise. Delayed onset muscle soreness (DOMS) peaks at around 24 h. Lower panel: Polymorphonuclear leukocytes (PMN) begin infiltrating the muscle immediately after the cessation of exercise. These differentiate into macrophages that ingest debris and apoptotic neutrophils. The production of local pro-inflammatory cytokines triggers the phenotypic switch from M1 macrophages to alternatively activated M2 macrophages. The early stage represents the inflammation phase, while the later stage indicates the beginning of the resolution phase. This figure is in part based on [42,51].

Aging is concomitant with alterations in the onset and duration of EIMD. The mechanisms of EIMD have been extensively reviewed elsewhere [41]. Known age-related dysregulations associated with EIMD entail changes regarding satellite cells, ECM composition, mitochondrial activity, inflammation, and lipid mediator production. Our focus in this review will be on changes in ECM and lipid mediators. Age-related changes in satellite cell activity have been reviewed extensively elsewhere and are thus only briefly mentioned. Several factors are pertinent during the recovery phase from exercise and engender age-related differences (Figure 1).

### 3.1. Difference in Recovery from Resistance Exercise in Aged Muscles

Age-related impairments in recovery from EIMD have been reported in animal studies [52,53,54]. Works on rodents show that older muscles are more susceptible to EIMD and require a longer recovery period [55,56,57,58]. The effect of age on RE has not reached a consensus in humans. It is generally agreed that individuals experience diminished physiological responses in advancing age. This is exemplified in exercise response by anabolic resistance, where there are reduced gains in muscle mass and strength following resistance training. However, in the scheme of repair and regeneration, the degree of impairment is less clear. The mechanistic gap of age-related impairments is obvious, and while previous studies have addressed multiple variables [12,59,60,61,62], this tenet is only supported by some studies, while a body of contradictory evidence also exists [59,63,64].

While there are still gaps in our understanding, and randomized controlled trials that comprehensively address multiple variables are lacking, several studies provide (indirect) insights into the altered response of aged muscle to RE. A decrease in the ability of the muscle to be excited at rest may be a peripheral alteration with age that increases stress on fewer muscle fibers, predisposing older individuals to greater damage [65]. In a comparison study between trained young and middle-aged males, as well as untrained middle-aged males, it was concluded that the level of damage after a squatting exercise was greatest in the order of untrained middle-age, trained middle-age, then young trained males [17]. Here, the results hint that training status may benefit older athletes in recovery, but the impacts of external therapeutic applications (i.e., nutrition and exercise) are out of the scope of this review. Similarly, in another subset of trained young and old men, the older group maintained lower leg strength from day 0 to day 4 following eccentric exercise compared to the young group. Although similar recovery was seen in both muscle function and DOMS [66]. Quantitatively, using creatine kinase as the primary parameter, older subjects demonstrated higher levels of damage markers after three 15 min bouts of eccentric exercise using a cycle ergometer [67]. Weaker, fewer, and less excitable muscles are just a few characteristics that may result in greater damage in older adults. On the contrary, these traits may also contribute to less damage, due to decreased performance capabilities, as it was reported that older men were not more susceptible to muscle damage after eccentric exercise of the biceps brachii [59]. In fact, greater damage was even observed in younger men. This may be explained by the similar muscle mass between the two groups and the greater preservation of muscle mass and strength in the upper body [59]. The same was seen in the gastrocnemius. After 150 eccentric contractions, no difference was reported between younger and older adults. It should be noted that the gastrocnemius is composed of a greater proportion of slow-twitch type 1 fibers compared to the commonly evaluated vastus lateralis [68]. With age, muscle fiber composition is skewed towards type 1, meaning there is generally a smaller difference between high type 1 fiber composed muscles between younger and older individuals. Additionally, during the early post-recovery period, the increase in Pax7+ cells was similar between young and older men at 48 h. However, this similarity was absent in type II fibers [69]. This disparity suggest that the fiber type composition of the muscle may determine the rate of decline in observed age-related differences, with muscle groups that are predominantly type II being more susceptible. Nevertheless, in the vastus lateralis, Sorenson et al. also found that older muscle was less susceptible to eccentric contraction-induced muscle damage [64]. The authors purported this to be a cause of the high training status of their older population as evidenced by a lack of difference in myofiber size between the young and old group. Though generally, older adults display a smaller myofiber size, perhaps due to factors that impair satellite cell behavior [70].

In addition, alterations in muscle protein turnover in older age are known. The balance between muscle protein synthesis (MPS) and breakdown (MPB) determines whether new contractile protein mass can be accrued, and functional muscle mass be gained. An acute bout of RE is known to increase MPS rates by ~100% in untrained individuals [71]. Interestingly, changes to MPS after an acute RE bout only poorly correlate with long-term gains in muscle mass [72]. This appears to be the case because the first exercise bouts are associated with a disproportionate amount of muscle damage, and after this muscle damage subsides, correlations between MPS and long-term hypertrophy become remarkably high [73]. It is likely that the early increase in MPS is repair-oriented, while MPS contributes to hypertrophy only in later stages. This suggests that measuring MPS does not necessarily reflect net protein accrual, but rather the remodeling state of muscle. In agreement with this, it has been shown in rodents that MPS and associated anabolic signals can be increased in scenarios where overall muscle mass is lost rather than gained [74,75,76,77]. Indeed, pre-clinical and clinical studies suggest that the amount of muscle damage in response to RE might be more pronounced in older compared to younger individuals [56,78]. Thus, MPS might be the sum of ongoing remodeling and muscle damage as observations reported a trend for higher basal MPS rates in individuals with sarcopenia compared to healthy older adults [79]. Differences in MPS rates likely reflect longer required periods of active remodeling prior to MPS contributions to hypertrophy, adding another explanation to why less hypertrophy may be seen in old individuals.

In line with this idea that anabolic processes and signals may be hallmarks of cellular distress in certain scenarios, such as acute exercise and aging, are reports that found increased activity of mTORC1 in older rodents [75,80]. Inhibiting those signals resulted in a delay of sarcopenia and improved muscle function and size [75,80]. These observations also align with the finding that MPS and anabolic signals in response to RE in young individuals decrease rapidly in response to chronic exercise [81,82,83], and that long-term improvements in muscle mass correlate with a decrease in mTORC1 signaling rather than an increase [84]. Indeed, older individuals tend to have higher baseline mTORC1 signaling than young individuals in response to chronic training but are unable to further increase it as an adaptive response to late RE bouts in conjunction with protein intake [85]. These discrepancies between young and old muscle, and the fact that the latter appears to be in a “damaged-like” state that requires constant remodeling, may explain part of the anabolic resistance and prolonged recovery times to achieve the same results as their younger counterparts [86].

### 3.2. Satellite Cell Contribution to Decreased Hypertrophic Potential

Satellite cells are the foundation of muscle fiber regenerative capacity [87] and the maintenance of muscle mass [88]. Quiescent satellite cells lay between the muscle fiber and basal lamina. One stimulus of satellite cell mitotic activity is exercise. Following high-intensity exercise, satellite cell activity can be seen as early as four days following the exercise [89]. This elevated level of satellite cells is maintained until the muscle is no longer subjected to training [90]. Satellite cell response can be adapted to chronic resistance training and plays a primary role in enhancing muscle size. A 16-week training regimen was shown to elicit greater satellite cell response compared to a single strength training session [91]. Therefore, satellite cells play an important role in regulating the long-term modulation of muscle fiber size.

This hypertrophic potential of muscle decreases with age. Old muscles do not hypertrophy to the same extent as young muscles when exposed to an identical stimulus [92,93,94]. But it is still a topic of debate whether its origins arise from a reduction in the number of muscle satellite cells or only the proliferative potential. In mice, both a reduction and no difference have been reported [95,96]. Snijders et al. demonstrated that age can impact satellite cell activation [97]. They compared the response of younger and older subjects to a single bout of resistance-based exercise. The satellite cell response in type II fibers was significantly delayed in older subjects, whereas satellite cell content was similar. Therefore, it seems that it is not so much decreased cell content but changes in the satellite cell niche that disrupts the regeneration response [98]. The insulin-like growth factor-1 (IGF-1) family may be one likely culprit. An exaggerated and dysregulated IGF-1 response was concomitant with lower and delayed satellite cell expression in older muscle [99]. The IGF-1 family may regulate myostatin and suppress satellite cell proliferation, which has been previously seen in older humans [100]. The satellite cell niche continues to be implicated as contributing to declines in skeletal muscle regenerative potential. Previous works have shown that satellite cell function can be fully rejuvenated when seeded on a characteristically younger substrate [101]. An important contributor to this niche is the ECM.

### 3.3. Transition of the ECM to a Fibrogenic Phenotype impacts Recovery

The skeletal muscle ECM is a three-dimensional network of collagenous components and macromolecules (i.e., glycoproteins, proteoglycans) that facilitates structure support, cell-to-cell communication, and the regulation of muscle stem cells [39,102,103,104,105]. The ECM is composed of three layers, the epimysium which encases the whole muscle, the perimysium surrounding the fascicles (grouping of muscle fibers), and the endomysium around each muscle fiber [106]. The endomysium includes the basal laminae and reticular laminae [107]. More recently, it has been increasingly recognized that the ECM is a dynamic player during reparative adaptations. With age, there is the simultaneous emergence of a fibrotic phenotype as a result of the decreased turnover of collagens [108]. The consequence manifests as an excessive accumulation of intramuscular connective tissue, an increase in non-enzymatic crosslinking, and an increased half-life of collagens [109,110,111]. These age-associated changes contribute to increased muscle stiffness, reduced strength, and heightened vulnerability to injury in muscles. A careful balance between the synthesis and degradation of collagens maintains optimal ECM conditions. Matrix metalloproteinases (MMP) degrade collagens. MMP-2 and MMP-9 are the most implicated in skeletal muscle. In old age, MMP-2 and MMP-9 lose their responsiveness to exercise-initiated ECM breakdown [112]. This is the likely culprit of decreased collagen turnover and ECM remodeling. MMP-2 and -9 are the major MMPs for the breakdown of collagen IV and V, the main component of the basal lamina/basement membrane, as well as other macromolecules such as fibronectin, proteoglycans, and laminin [113,114]. One feature of the basal lamina that changes with age is its progressive thickening, a likely result of decreased protease activity. Since satellite cells must cross this barrier to enter the muscle, its thickening may limit satellite cell differentiation and proliferation for muscle regeneration. Moreover, the inhibitor of MMPs, tissue inhibitors of metalloproteinases-1/2 (TIMP-1/2), is increased in the gene expression profile of aged animal fibroblasts. Enhanced inhibitory activity of MMPs likely supports ECM accumulation and the ensuing fibrotic phenotype [115].

As mentioned above, the dynamic interplay between satellite cells and their microenvironment is recognized to play a role in directing resident cell function. The ECM governs the microenvironment surrounding the satellite cells by providing mechanical and structural cues [116,117]. Cells residing in the ECM release a myriad of molecules, including insulin-like growth factor and fibroblast growth factor. These growth factors are key signaling molecules that orchestrate the various stages of satellite cell function. Satellite cells are highly sensitive to changes in the niche, and aging of the ECM concomitantly drives satellite cells towards a fibrogenic lineage [115]. Aged skeletal muscle displays a change in the composition and properties of collagen, specifically a decrease in collagen tortuosity, the degree of anisotrophy (or organization), and the abundance of compliant ECM components (i.e., elastin, collagen type III). This is all in accordance with the increased stiffness seen in aged muscle. The upregulation of advanced glycation end-products (AGEs) in aging skeletal muscle tissues may also contribute to stiffness. AGEs preferentially accrue on collagens due to their long half-life. Non-enzymatic cross-linking by AGEs decreases susceptibility to degradation by MMPs, causing collagen build-up [106]. The receptor for AGE, RAGE, is upregulated after acute injury and sarcopenic conditions [118]. Signaling potentiated by the AGE–RAGE pathway can stimulate the phosphorylation of NFkB and elevate p-38 MAPK signaling [119,120]. NFkB is implicated as a factor in the increase in collagen content and proinflammatory cytokine, whereas p-38 MAPK may disrupt satellite cell signaling. In vitro seeding of satellite cells on a stiff substrate decreased stemness, promoting fibrogenic conversion [101]. The stiffening of the muscle may be one reason for the dysfunction seen in satellite cells’ proliferative capacity. The stiffness of the ECM may also feed back onto itself to further increase secretions from fibroblasts. Cytoskeletal tension of neighboring cells is increased with stiffness, and in isolated aged animal muscle, the change in force transmission along the cytoskeleton modulates nuclear morphology and subsequent cellular gene expression. Furthermore, there is an increase in the nuclear translocation of YAZ/TAZ, the transcriptional regulators that relay mechanical signals imposed by the matrix which modulates fibroblast numbers and matrix protein secretions, including those making up the transitional matrix [115]. The upregulation of a “transitional matrix” is less researched. First coined by Calve et al., the transient appearance of the glycoproteins fibronectin and tenascin-C and hyaluronic acid provides important regenerative cues [121]. This matrix is upregulated following muscle damage in young humans [64,122]. Conversely, this appearance was blunted in older humans [64]. Similarly, Lukjanenko et al. found a loss of fibronectin in the aged stem cell niche of mouse skeletal muscle [101]. Fibronectin predominantly functions to support an adhesive environment for the differentiation of myoblasts [121,123]. It also participates in physically directing collagen and satellite cells as it is the preferred adhesion substrate through integrins embedded in the myofiber membrane [101,124,125]. Thus, a depletion of fibronectin would deregulate satellite cells due to insufficient attachment. A loss of tenascin-c, a mechano-regulated ECM protein, may prevent cell motility and proliferation [122,126]. Contrary to fibronectin, tenascin-c facilitates a de-adhesive environment that relieves substrates adhered to the ECM. It seems that the temporal coordination of its release may be crucial in directing satellite cell behavior; however, further research is required to access the mechanistic relationship between these two matrix components.

### 3.4. Mitochondrial Dysfunction and Implications on Oxidative Stress and Inflammation

There are significant age-related changes in the mitochondria in skeletal muscle. Not only does the morphology change as mitochondria become enlarged and rounded, there is a loss of mitochondria content and reduced enzymatic activity, protein markers, and mitochondrial DNA [127]. Mitochondrial morphology has been linked to the pathogenesis of DOMS [127]. Mitochondria are a major source of oxidative stress and inflammation through the production of reactive oxygen species and damage-associated molecular patterns (DAMPs), respectively. A recent study using a bioinformatic approach confirmed that skeletal muscle mitochondrial damage causes the accumulation of inflammatory factors in DOMS [128]. TNFa, a hallmark and regulator of systemic inflammation, which is also found to be elevated in plasma of subjects experiencing DOMS, increases linearly with inflammatory activity in the skeletal muscle [129]. Moreover, mitochondrial dysfunction is observed in aging and is thought to be closely related to sarcopenia. Compromised mitochondrial health has many impacts on muscle repair. Since ATP is of high demand during myogenesis, reduced ATP generation will limit the extent of repair [130]. There also seems to be an association between mitochondrial health and myoblast proliferation, differentiation, and migration [131]. Since glucose is utilized as the primary substrate for oxidative phosphorylation in differentiating satellite cells [132], disruption in mitochondrial bioenergetics or reduced oxidative capacity can block satellite cell differentiation [133]. This emphasizes the importance of healthy mitochondria in satellite cell proliferation and differentiation. Inadequate mitochondrial autophagy (mitophagy) can impact myoblast differentiation and potentially activate the inflammatory process through the release of DAMPs or, in part, through NLRP3 [134,135,136].

### 3.5. Inflammatory Changes Post-Exercise

Following a damaging bout of unaccustomed exercise, transient inflammation is essential for muscle repair following injury. The timely resolution of this acute-phase inflammation is crucial for long-term beneficial exercise adaptation and the preservation of healthy tissue [137]. Aging is associated with an increase in inflammation, even in the absence of disease, termed inflammaging [138]. Elevated levels of pro-inflammatory proteins, such as IL-6, TNFa, and C-reactive protein, are typically seen in higher age [139]. Chronic low-grade inflammation lies at the nexus of many age-related impairments and chronic diseases including obesity, cardiovascular disease, and diabetes [140,141,142]. Some proposed causes of inflammation include declines in immune function, age-related chronic diseases [143], increased oxidative stress, and compromised antioxidant capacity [144]. However, the multifactorial nature of inflammation renders it difficult to delineate whether its origins are a cause or consequence. Basal low-grade chronic inflammation may interfere with acute-phase inflammation by mounting an exaggerated response that is unable to be completely resolved. Over time, this sets in motion a detrimental cycle of persistent inflammation from past exposures, perpetually amplifying subsequent responses and resulting in a permanent alteration of the basal immune profile [145]. But the blunting of this acute inflammatory response may impede beneficial skeletal muscle adaptations to exercise [146]. The respective literature on the effects of using anti-inflammatory drugs for EIMD is yet unclear [35,147]. Patients of a geriatric hospital with acute inflammation exhibited reduced inflammation-induced muscle weakness and improved performance [147]. However, robust evidence is lacking. As previously reported by Peake et al., older adults seem to exhibit delayed repair and regeneration of muscle damage [14]. During the immediate period, older adults are capable of cumulating adequate, but greater, pro-inflammatory signals (i.e., suppressor of cytokine signaling 3, IL-6, TNFa, IL-8) [148,149]. In the days following exercise damage, age appears to hinder the recruitment of macrophages [64,89]. Monocyte chemoattractant protein-1 (MCP-1), an important chemoattractant, displays delayed kinetics old humans, peaking at 24 h compared to the 3 h peak observed in their younger counterparts after 300 maximal eccentric contractions [64]. In contrast to their younger counterparts, older individuals reveal impaired macrophage infiltration at both 24 and 72 h [148,149]. Intriguingly, this impairment is accompanied by a continued increase in macrophage presence 4–7 days post-exercise, a period when macrophage numbers have already reverted to basal levels in the young [150]. The prolonged inflammatory phase in higher age may be a sign of failure to resolve and contribute to the already present systemic inflammation.

### 3.6. Lipid Mediator Involvement on Resolution of Inflammation

It is now recognized that the resolution of inflammation is an active process, and not a passive waning of pro-inflammatory signals [150]. As alluded to previously, lipid mediators play an important role in mediating local and systemic inflammatory responses [51]. At basal conditions, these mediators sourced from polyunsaturated fatty acids (PUFAs) primarily coming from the diet are suspended as phospholipids in the cell membrane. When they are required, rapid liberation by phospholipases (i.e., PLA2) allows subsequent conversion into lipid mediators [151]. The oxidation of the mobilized PUFA substrate can occur through three major pathways: (1) cyclooxygenase (COX), (2) lipoxygenase (LOX), and (3) epoxygenase—catalyzed by cytochrome P450 (CYP). Through these pathways, hundreds of lipid mediators can be synthesized [51]. The early stages of tissue injury are dominated by pro-inflammatory lipid mediators. What is known as the hallmark manifestations of inflammation (calor, rubor, dolor, and tumor) is driven by classical pro-inflammatory lipid mediators, prostaglandins, and leukotrienes. These are the initial lipid mediators that appear. In the later stages, a shift towards anti-inflammatory and pro-resolving mediators is seen (e.g., lipoxins, resolvins, protectins, and maresins) [152]. These lipid mediators are collectively termed specialized pro-resolving lipid mediators (SPMs) and are important signaling molecules within skeletal muscle [153]. They play an important role in restoring muscle homeostasis by limiting polymorphonuclear leukocyte (PMN) infiltration and promoting clearance by mediating macrophage polarization towards an M2 phenotype or resolution-phase (rM) phenotype [152]. Temporal changes in lipid mediators occur in peripheral blood during post-exercise recovery. To date, there has only been one study published that uses a metabolipidomic approach to profile PUFA metabolites in response to unaccustomed exercise [154]. Several lipid mediators with anti-inflammatory and pro-resolving properties were detected following an eccentric exercise bout, including arachidonic acid-derived lipoxins (LXA4 and LXB4), eicosapentaenoic acid (E-series) and docosahexaenoic acid (D-series)-derived resolvins (RvD1 and RvE1), and protectins (PD1 isomer 10S, 17S-diHDoHE). During aging, there appears to be a deficit in SPM production and/or response. Older mice that exhibited an inflammatory cytokine and lipid mediator profile at baseline, evidenced by higher levels of pro-inflammatory cytokine, prostaglandin, and thromboxane levels, respectively, demonstrated delayed resolution of acute inflammation [151]. Following injection with zymosan to induce peritonitis, older mice had a resolution interval that was 85% higher (defined as the time between maximal and 50% maximal influx of neutrophils). They also displayed exacerbated PMN recruitment, a higher total number of monocytes and macrophages prior to and during the resolution phase, and impaired uptake of apoptotic PMN [155]. Temporal SPM levels were dysregulated with a later appearance of pro-resolving mediators (incl. RvD1). This suggests an age-related impairment in the resolution phase. The temporal changes observed during post-exercise recovery provide valuable insights into skeletal muscle homeostasis, but further research must be conducted to further delineate the differences.

## 4. Future Directions

The duration of the follow-up in the majority of studies is limited to the initial 24 to 72 h, making it difficult to distinguish the timeline to complete recovery. Additionally, maximal eccentric exercises, while employed in studies, may not authentically mirror real-world activities. Instead, conventional RE protocols consisting of both concentric and eccentric contractions should be utilized to have greater application, especially to older individuals. In addition, future investigations should pivot towards associating primary outcomes with functional parameters and molecular changes to pinpoint potential drivers of age-related deterioration. Although invasive, muscle biopsies should be optimized across multiple time points to accurately identify the endpoint of local repair. Greater precautions should be taken to match exercise intensity between age groups as older adults generally produce less force and power. Thus, exercise protocols should take relative rather than absolute differences into account. The majority of studies comparing recovery from EIMD involve older adults with already high fitness levels as evidenced by similar muscle mass. Critically, future studies should diversify participant recruitment by targeting older adults displaying age-related deficits, discerned through comprehensive assessments such as muscle function testing, body composition, or skeletal muscle fiber-typing. Additionally, many older adults included in the studies only averaged around 65 years of age. At this age, individuals may not yet be exhibiting the detrimental effects of aging. Instead, to understand the specific mechanisms impacting recovery, perhaps an older population showing signs of sarcopenia would elucidate greater differences from younger populations.

## 5. Conclusions

**Figure 2:**
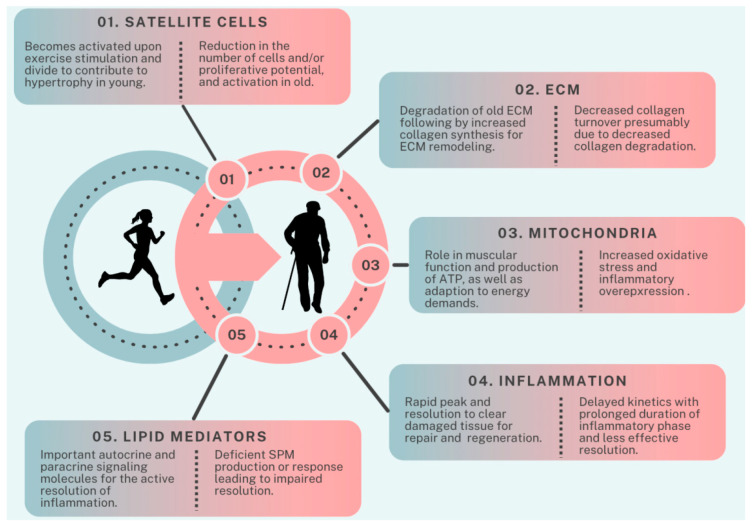
Factors contributing to age-related differences in recovery from resistance exercise. ECM: extracellular matrix, SPM: specialized pro-resolving lipid mediators.

In conclusion, aging skeletal muscles undergo structural and functional changes contributing to physical limitations and increased health risks. Although RE proves effective in counteracting these deteriorations, the introduction of novel exercises to untrained muscles can disrupt the delicate balance of muscle homeostasis, resulting in exercise-induced muscle damage. Successful recovery from EIMD relies on a precisely orchestrated sequence of cellular events, crucial for achieving cumulative positive effects through RE. Alterations of factors like satellite cells, extracellular matrix remodeling, mitochondria, inflammation, and lipid mediators contribute to delayed recovery in older individuals (Figure 2). Understanding these complexities is crucial for effective exercise, trauma recovery, and rehabilitation strategies across the lifespan. Future research should refine the methodologies, ensure relative exercise intensity, and diversify participant recruitment for a nuanced exploration of aging muscle recovery mechanisms.
