# Correction: Li et al. Age-Associated Differences in Recovery from Exercise-Induced Muscle Damage. *Cells* 2024, *13*, 255

**DOI:** 10.3390/cells14191564

**Published:** 2025-10-09

**Authors:** Donna Ching Wah Li, Stefan Rudloff, Henning Tim Langer, Kristina Norman, Catrin Herpich

**Affiliations:** 1 Department of Nutrition and Gerontology, German Institute of Human Nutrition Potsdam-Rehbrücke, 14558 Nuthetal, Germany; 2 Institute of Nutritional Science, University of Potsdam, 14558 Nuthetal, Germany; 3 Department of Geriatrics and Medical Gerontology, Charité–Universitätsmedizin Berlin, Corporate Member of Freie Universität Berlin and Humboldt-Universität zu Berlin, 13347 Berlin, Germany; 4 Department of Medicine, Weill Cornell Medicine, New York, NY 10065, USA; 5 German Center for Cardiovascular Research (DZHK), Partner Site Berlin, 10785 Berlin, Germany

## Error in Figure

In the original publication [1], there was a mistake in Figure 1 as published. The figure was changed during the revision process. An old figure was uploaded by mistake, which did not indicate the differences between older and younger adults. The corrected version of Figure 1 appears below. The authors state that the scientific conclusions are unaffected. This correction was approved by the Academic Editor. The original publication has also been updated.

## Figures and Tables

**Figure 1:**
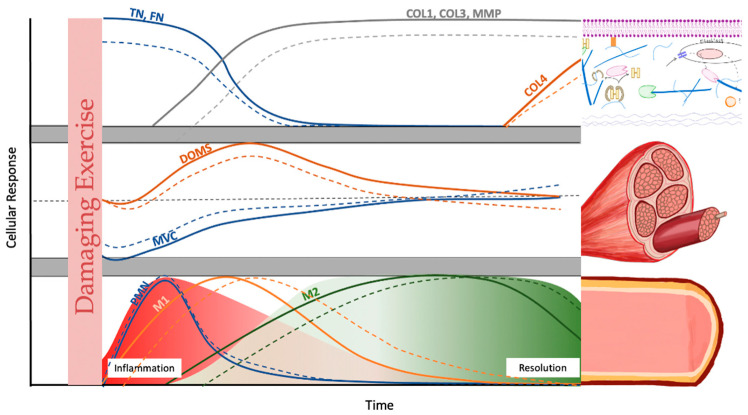
Schematic comparison of the cellular response kinetics of post damaging exercise in young (line) and old humans (dotted line): extracellular matrix (ECM) changes (upper panel), indirect markers (middle panel), and inflammatory expression (lower panel). Upper panel: Tenascin-C (TN) and fibronectin (FN) are part of the transitional matrix and are thought to direct early satellite cell movement. Subsequently, collagens (COL) 1 and 3 and their matrix metalloproteinases (MMPs) increase for ECM remodeling. COL 4 is, an important component of the basement membrane, is thought to be remodeled during later stages. Middle panel: Decreases in maximal voluntary contraction (MVC) are prevalent immediately following exercise. Delayed onset muscle soreness (DOMS) peaks at around 24 h. Lower panel: Polymorphonuclear leukocytes (PMN) begin infiltrating the muscle immediately after the cessation of exercise. These differentiate into macrophages that ingest debris and apoptotic neutrophils. The production of local pro-inflammatory cytokines triggers the phenotypic switch from M1 macrophages to alternatively activated M2 macrophages. The early stage represents the inflammation phase, while the later stage indicates the beginning of the resolution phase. This figure is in part based on [42,51].
